# Financial capacity in frontotemporal dementia and related presentations

**DOI:** 10.1007/s00415-019-09317-w

**Published:** 2019-04-22

**Authors:** Sascha Gill, Mervin Blair, Mavis Kershaw, Sarah Jesso, Julia MacKinley, Kristy Coleman, Koula Pantazopoulos, Stephen Pasternak, Elizabeth Finger

**Affiliations:** 1 Department of Clinical Neurological Sciences, Schulich School of Medicine and Dentistry, Parkwood Institute, Lawson Health Research Institute, University of Western Ontario, 550 Wellington Rd, London, ON Canada; 2 Australian Psychological Society, Melbourne, Australia; 3 International Association of Applied Psychology, New York, USA; 4 Australia and New Zealand Association of Psychiatry, Psychology and Law, Melbourne, Australia

**Keywords:** Financial capacity, Frontotemporal dementia, FTD phenocopy

## Abstract

**Background:**

Changes in financial judgement and skills can herald a neurodegenerative dementia and are a common reason for referral for cognitive neurologic assessment. However, patients with neurodegenerative diseases affecting the frontal or temporal lobes may perform well on standard cognitive tests, complicating clinical determinations about their diagnosis and financial capacity.

**Methods:**

Forty-five patients with possible or probable FTD or Alzheimer’s disease and 22 healthy controls completed two financial assessment batteries, the FACT and the FCAI. Patients’ performance was compared to study partner estimates of patients’ financial abilities.

**Results:**

All three patient groups performed worse than controls on both the FACT and the FCAI. Study partners over-estimated the performance of patients with Alzheimer’s disease.

**Conclusions:**

These initial findings suggest that accurate clinical assessment of financial skills and judgement in patients with possible neurodegenerative dementias requires performance-based assessment.

**Electronic supplementary material:**

The online version of this article (10.1007/s00415-019-09317-w) contains supplementary material, which is available to authorized users.

## Introduction

Financial capacity is defined as the ability to manage one’s financial affairs consistent with personal self-interest [1]. It represents a cognitively complex process involving declarative and procedural knowledge and is vulnerable to neurological sequelae, including neurodegenerative diseases [2]. Financial deficits are often identified by family members while patients with dementia tend to be unaware of their declining financial abilities [3]. In a clinical setting, identification of impaired financial capacity may provide evidence of early functional changes associated with dementia. The management of such patients requires that physicians make subjective decisions that may limit patients’ financial autonomy, or alternatively leave patients and their families financially vulnerable (falling for financial scams, spending into debt). Despite its importance, financial capacity has received little attention, specifically in the non-Alzheimer’s dementia literature, with no current gold standard method of evaluation.

Research shows that patients with Alzheimer’s disease (AD) and mild cognitive impairment (MCI) exhibit financial capacity deficits [4, 5]. Using a performance-based financial measure, it was identified that financial skills decline with increasing severity of AD [1]. Although these studies provide evidence for impaired financial abilities in Alzheimer’s disease, it is unclear the extent to which financial capacity is compromised in other non-Alzheimer’s dementias, specifically early-onset dementias such as frontotemporal degeneration.

Behavioural variant-frontotemporal degeneration (bvFTD) is primarily characterized by behavioural changes such as disinhibition, lack of insight, decreased impulse control and cognitive deficits involving attention and executive functions (e.g., planning, reasoning, judgement, problem solving, sequencing, mental flexibility, and risky decision making) [6–10]. While financial capacity involves relatively simple skills such as recognition of coins/bills, attention tracking, and arithmetic skills [11, 12], it also involves more higher-level abilities that are likely relevant to bvFTD, such as conceptual, pragmatic, and judgement abilities [13]. Patients with FTD may accrue large debts due to changes in spending and poor financial judgment. In retrospect, family members often cite a significant financial loss as one of the first signs of cognitive or behavioural changes in FTD [14]. Presently, research indicating financial difficulties in FTD including bvFTD is limited, often restricted to subjective reporting methods. For instance, Chiong et al. [7], conducted a retrospective chart review and observed that financial skills (e.g., spending excessively, paying less attention to losses) were affected in bvFTD. However, the authors noted that sole reliance on clinical histories based on caregiver reports likely limited their results. Moreover, no research has examined financial skills in patients with possible bvFTD who show clinical symptoms of bvFTD but lack imaging or genetic confirmation, or in so-called ‘phenocopy’ cases of FTD, who present with bvFTD-like clinical symptomatology which often includes changes in financial judgment, but have relatively preserved cognitive skills, with no evidence of neuroimaging abnormalities over time [7, 15]. Such patients who may be referred for frontal-type behaviours but may not meet criteria for probable bvFTD represent a particular challenge for clinical management, as they do not have a confirmed diagnosis of dementia at time of presentation or initial evaluation, but may display early and significant deficits in financial skills.

Given the limited research on financial skills in bvFTD, particularly using performance-based measures, we aimed to examine whether financial capacity is affected in this population and investigate the extent to which patients’ financial abilities were consistent with family reports. We also sought to explore the performance of patients in which changes in financial judgement or investment success were among the chief or early symptoms reported by caregivers, but who did not meet criteria for bvFTD, AD or vascular cognitive impairment at time of initial assessment, identified as phenocopy or possible bvFTD patients (pheno/poss-bvFTD). In this study, two combined self-report and performance-based measures of financial capacity were used to (1) compare bvFTD patients to healthy controls, AD patients, and pheno/poss-bvFTD patients, and (2) examine whether third-party reports were comparable to patients’ results. The two financial measures used were validated in older adults, accessible and feasible to address our research questions [16, 17]; additionally, one of the two measures allowed for third-party evaluation on patient financial capacity. Due to impaired executive ability, we hypothesized that bvFTD patients would show poor financial skills relative to controls. Comparison of bvFTD to AD and pheno/poss-bvFTD patients was exploratory. In addition, because of reduced insight and difficulties with poor judgement and impulse control in bvFTD patients, we predicted discrepancy in family reports of patients’ abilities relative to patients’ self-reports and performance on financial measures.

## Methods

### Participants

Participants in clinical groups were recruited from the Cognitive Neurology and Alzheimer’s Disease Research Centre at Parkwood Institute and were also identified in the cognitive neurology clinic database if they met study criteria. Initial clinical assessment of patients included a detailed history and neurologic examination by a behavioural neurologist, cognitive testing which included the MMSE, MoCA, Clock draw test (including copy), Immediate and Delayed paragraph recall adapted for Canadian patients from the Rivermead Test, letter and animal fluency, naming from the 15-item Boston Naming Test and/or Western Aphasia Battery, Trails A and B, and a depression inventory (Beck Depression Inventory for patients < 65 years old, Geriatric Depression Scale for patients over age 65), and review of prior neuroimaging which included a CT head, MRI head, and/or SPECT scan. While genetics were not available at time of presentation, where indicated based on age of onset and family history, subsequent genetic testing for dominant mutations causing FTD or early onset AD was completed. Due to the unavailability of clinical PET scans or CSF amyloid and tau analysis for dementia in Ontario, these assessments were not performed. Forty-five patients and 22 healthy controls participated in the study: *n* = 15 bvFTD, *n* = 15 patients with AD, and *n* = 15 initially referred for question of bvFTD, but whom did not meet criteria for probable bvFTD at time of the initial evaluation (pheno/poss-bvFTD). Eligible patients with bvFTD met the diagnostic criteria for behavioural variant FTD [18]. Pheno/poss-bvFTD patients represented a heterogenous group of individuals who did not meet criteria for bvFTD or AD at the time of referral, and instead met criteria for possible behavioural variant FTD at their initial evaluation [18]. Overtime, patients in this group were diagnosed with possible bvFTD or phenocopy bvFTD, or other related neurologic disorders, as detailed in Supplementary Table 1. Patients with AD were diagnosed according to the National Institute of Neurological and Communicative Disorders and Stroke and the Alzheimer’s Disease and Related Disorders Association (NINCDS-ADRDA) [19]. All patients scored ten or higher on the Montreal Cognitive Assessment (MOCA [20]). All patients included were accompanied by a study partner willing to participate in the study. The study partner was either a spouse, an adult–child or other close relative who interacted with the patient on a regular basis (AD patients: 87% spouse, 13% adult–child; bvFTD patients: 67% spouse, 27% adult–child, 6% other relative; pheno/poss-bvFTD: 83% spouse, 17% adult–child). Based on general clinical evaluations, patients with comprehension deficits or language impairments that would interfere with test completion were excluded. Other exclusion criteria included known history of stroke, traumatic brain injury, and a history of other neurologic or psychiatric disorder that could account for the patient’s symptoms. Forty-five participants that were deemed testable by clinical staff based on clinical evaluation and notes were included in the study and all were able to complete study measures.

Twenty-two healthy control volunteers were recruited from the community through advertisements or approached while accompanying friends or family to clinic visits. Healthy volunteers were included if they reported general good physical and cognitive health, and excluded if they reported medical, psychological, or motor conditions that impacted their activities of daily living or their performance on cognitive tests. Based on this criteria, 20 volunteers who met study criteria were included in this study. Although self-report rather than test/questionnaire score was the primary basis for inclusion in the study, two volunteers were excluded due to extreme test/questionnaire score and clinical observation: one had a MOCA score of 18 (indicative of cognitive impairment and consistent with clinical observations) and one had a Beck FAST screen score of 8 (indicative of depressive symptomatology and consistent with clinical observations).

This study was approved by the Health Sciences Research Ethics Board at Western University, London, Ontario, Canada. The STROBE guidelines were used in the reporting of the results.

## Measures

### Financial assessments

Financial Assessment and Capacity Test (FACT): is a validated measure to assess current financial decision-making capacity in elderly individuals [16]. Using Appelbaum’s conceptual model [21] with four conditions as a foundation, the FACT includes items that evaluate decision-making capacity such as ability to communicate choices, to understand relevant information, to comprehend risks, and to rationally manipulate information [21, 22]. It is administered in a structured interview format and consists of 46 items. The FACT is divided into multiple domains that are related to Applebaum’s model, which include memory (e.g., repeating items on a grocery list), reading/writing, calculation/attention (e.g., counting and calculating bills), daily financial tasks (e.g., bill identification, payment, budgeting), general financial knowledge (e.g., identifying the importance of bank accounts, savings, taxes and having a will), understanding assets (e.g., providing examples of assets that a person could have), financial insight (e.g., identifying strengths/weaknesses in handling money, difficulty in managing finance, and level of happiness with one’s current finances), financial confidence (e.g., identifying current money problems) and rational beliefs about money (e.g., buying things one does not need, thoughts about others stealing one’s money). The test is typically completed in 30–40 min.

Financial Competence Assessment Inventory (FCAI): is a reliable and validated measure used to assess current financial competence [17]. It is administered in a structured interview format and consists of 41 items, with a combination of questions and functional tasks assessing financial abilities. Items from the FCAI are coded and scored in two ways: (i) six subscales/domains which include everyday financial abilities (e.g., awareness of bills), financial judgement (e.g., statement of long term financial goals), estate management (e.g., understanding of Power of Attorney), cognitive functioning related to financial tasks (e.g., calculation of account balance), debt management (e.g., identifying recent disconnection of household services due to non-payment) and support resources (e.g., knowledge of community services/government programs) (referred to as FCAI-6 in this study); and (ii) recoded into four subscales which were based on Applebaumm and Grisso’s legal criteria [21] and are classified into domains of understanding (i.e., ability to identify and comprehend the concepts involved in making a decision), appreciation (i.e., ability to think in an abstract manner about the situation and implications of a decision), reasoning (i.e., ability to apply logic and weigh risks and benefits of a decision) and expressing a choice (i.e., ability to decide between two or more options and convey a decision) (referred to as FCAI-4 in this study) (see [17], for details on each scale). Two versions of this FCAI were used in this study, a patient version [17] and a third-party version [23], the latter of which was administered to study partners with the instruction to rate/report their perception of the patient’s financial abilities for each item on the measure. The test is typically completed in 20 min with patients and 15 min with third parties.

## Procedure

Eligible participants were invited to the cognitive neurology clinic for a 2–3.5 h study session. Participants were first administered the MoCA [20] and other cognitive screening measures as part of a standard brief cognitive battery: Verbal Fluency test (FAS, animals) [24], Digit Span test [25], Digit Symbol Substitution test [25], Trail Making test [24], Stroop test [24], Western Aphasia Battery Comprehension subtests (WAB) [26], Neuropsychiatric Inventory (NPI) [27], and Lawton Instrumental Activities of Daily Living Scale (Lawton IADL) [28]. We note that diagnostic criteria as detailed earlier were used to diagnose or differentiate clinical syndromes and not the aforementioned cognitive test battery.

Trained research staff administered all cognitive and financial measures to the clinical patients and healthy volunteers. Study partners of clinical patients completed the third-party version of the FCAI in a separate room, either while the patient was being evaluated or within a month’s period.

### Statistical analysis

A series of one-way analyses of variance (ANOVAs) was conducted to examine demographic factors and cognitive test scores. Chi squared analysis was conducted to compare sex differences among groups.

Three multivariate analyses of variance (MANOVA) were conducted to assess FACT and FCAI scores among the groups (FTD, pheno/poss-bvFTD, AD, controls), respectively, followed by a series of univariate analysis of variance (ANOVA) on FACT and FCAI subscales. Post-hoc Bonferroni corrected contrasts were conducted to explore significant differences. Only FCAI standard score results are reported as the pattern of results replicated when raw scores were used. The percentages of participants in each group scoring in the mild impairment range or worse were calculated based on the FCAI total standard score of 100 and standard deviation of 15 (FCAI 2006 Matek Pty. Ltd.).

Group (FTD, pheno/poss-bvFTD, AD, controls) × FCAI-format (patient performance, third-party report) mixed-factorial ANOVAs were performed to identify any discrepancies between patient performance and third-party reports across the groups.

All statistical analyses were performed using the Statistical Package for the Social Sciences (SPSS version 24 for Windows, Chicago, IL, USA) and all hypotheses were tested at alpha level of 0.05 (two-tailed).

## Data availability

Raw data were generated at the Cognitive Neurology and Alzheimer’s Research Centre at Parkwood Institute. Derived data supporting the findings of this study are available from the corresponding author on request.

## Results

Table 1 shows demographic characteristics and cognitive test results of the groups. Mean age and scores on cognitive measures were significantly different across the groups using one-way ANOVAs, *p*s< 0.05. Post-hoc analysis revealed that bvFTD patients (*M* = 67.47, SD 8.66) were younger than AD patients (*M* = 77.33, SD 6.35), *p* < 0.05. Both groups had similarly low MoCA scores (FTD: *M* = 19.13, SD 4.67; AD: *M* = 17.40, SD 4.01), and each group scored lower on the MoCA compared to pheno/poss-bvFTD patients (*M* = 24.27, SD 3.41) and healthy controls (*M* = 27.25, SD 1.94), *p* < 0.05. This pattern of similarly poor scores by bvFTD and AD patients when compared to healthy controls was also evident across other cognitive measures, including semantic fluency, Digit Span, Digit Symbol, and the Stroop test, *p*s < 0.05 (see Table 1). These aforementioned results comparing the groups on cognitive tests were unchanged when age differences were accounted for using analysis of covariance (ANCOVA) with age serving as the covariate. The bvFTD group obtained a significantly higher score on the NPI than AD patients, but had a lower score on the Lawton IADL than pheno/poss-bvFTD patients, *p*s < 0.05 (see Table 1), indicating that bvFTD patients had more behavioural problems than AD patients and more functional difficulties than pheno/poss-bvFTD patients. Chi-squared analysis showed significant sex differences among the groups, *χ*^2^(3) = 9.0, *p *= 0.03, due to the low representation of women amongst pheno/poss-bvFTD patients compared to other groups.

**Table 1:** Demographic characteristics and cognitive test results

	AD (*n* = 15) *M* (SD)	bvFTD (*n* = 15) *M* (SD)	Pheno/poss-bvFTD (*n* = 15) *M* (SD)	Controls (*n* = 20) *M* (SD)	*F* value	*df*	*p* value	$\eta_{\text{p}}^{2}$
Age(years)	77.33 (6.35)	67.47 (8.66)	69.93 (6.55)	67.90 (8.93)	5.33	3, 61	.003^a^	0.21
Education(years)	14.20 (4.40)	13.93 (2.74)	14.00 (4.50	13.68 (2.91)	0.057	3, 60	0.982	0.003
Duration of illness (years)	5.143 (3.21)	5.04 (3.20)	6.70 (4.99)	–	0.826	2, 40	0.445	0.04
Gender (F:M)	5:10	8:7	2:13	12:8	–	3	0.03	
MoCA	17.40 (4.01)	19.13 (4.67)	24.27 (3.41)	27.25 (1.94)	28.078	3, 61	.000^b^	0.580
Semantic fluency	10.64 (3.23)	8.80 (3.84)	14.29 (4.51	19.90 (5.17)	22.25	3, 59	.000^c^	0.53
Phonemic fluency	31.53 (15.04)	18.67 (10.95)	31.08 (12.76)	42.30 (12.67)	9.58	3, 59	.000^d^	0.33
Digit span test	13.20 (4.26)	13.13 (4.29)	14.29 (3.69)	17.80 (4.19)	5.16	3, 60	.003^e^	0.21
Digit symbol test	32.27 (13.72)	39.64 (16.75)	43.92 (10.18)	68.30 (14.60)	21.95	3, 58	.000^f^	0.53
TMT-A	44.80 (24.82)	63.80 (37.16)	44.46 (13.94)	28.60 (8.77)	6.59	3, 59	.001^g^	0.25
TMT-B	164.47 (113.19)	186.64 (101.54)	186.62 (113.12)	73.55 (47.72)	5.86	3, 58	.001^h^	0.23
TMT-B minus A	119.67 (100.47)	127.79 (85.37)	146 (110.39)	44.95 (43.19)	4.79	3, 57	.005^i^	0.20
Stroop colour	20.60 (10.41)	17.50 (9.58)	25.50 (10.78)	37.32 (8.38)	13.69	3, 58	.000^j^	0.41
WAB—yes–no	59.20 (1.37)	60.00 (0.000)	59.60 (1.06)	59.70 (0.92)	1.73	3, 61	0.17	0.08
WAB—auditory word recognition	60.73 (3.13)	59.20 (1.42)	59.93 (0.26)	59.85 (0.37)	2.14	3, 61	0.10	0.10
WAB—sequential commands	84.00 (30.38)	76.47 (5.89)	77.47 (7.50)	79.60 (1.79)	0.72	3, 61	0.55	0.03
WAB—auditory verbal comprehension	184.47 (42. 61)	195.67 (5.95)	197.00 (7.41)	199.15 (2.21)	1.56	3, 61	0.21	0.07
WAB—comprehension of sentences	36.53 (5.83)	33.07 (6.04)	38.13 (4.17)	38.80 (2.93)	4.62	3, 61	.01^k^	0.19
WAB—reading commands	18.27 (4.89)	19.60 (0.74)	19.60 (1.30)	19.90 (0.31)	1.41	3, 61	0.25	0.07
NPI	12.8 (13.83)	30.67 (17.28)	26.5 (21.52)	–	4.16	2, 41	.02^l^	0.17
Lawton IADL (median/range)	4.0 (1–9)	2.0 (1–8)	5.0 (3–17)	–	4.26	2, 41	.02^m^	0.17

Gender analysis is based on Chi-squared test

All results indicated are based on univariate ANOVAs, *p*s<* 0*.05

*AD* Alzheimer’s disease, *F* female, *bvFTD* behavioural variant-frontotemporal dementia, *Lawton IADL* Lawton Instrumental Activities of Daily Living scale, *M* male, *M* mean, *MOCA* Montreal Cognitive Assessment, *NPI* Neuropsychiatric Inventory, *pheno/poss-bvFTD* phenocopy or possible behavioural variant-frontotemporal dementia, *SD* standard deviation, *TMT* trail-making test, *WAB* western aphasia battery

^a^AD were older than Controls and bvFTD

^b^Controls and pheno/poss-bvFTD scored higher than bvFTD and AD

^c^Controls scored higher than pheno/poss-bvFTD, bvFTD and AD; pheno/poss-bvFTD scored higher than bvFTD

^d^Controls scored higher than bvFTD; AD scored higher than bvFTD

^e^Controls scored higher than bvFTD and AD

^f^Controls scored higher than pheno/poss-bvFTD, bvFTD and AD

^g^Controls were more efficient (lower score) than bvFTD

^h^Controls were more efficient (lower score) than pheno/poss-bvFTD, bvFTD and AD

^i^Controls were more efficient (lower score) than pheno/poss-bvFTD, bvFTD

^j^Controls scored higher than pheno/poss-bvFTD, bvFTD and AD

^k^Controls and pheno/poss-bvFTD scored higher than bvFTD

^l^bvFTD scored higher than AD

^m^Pheno/poss-bvFTD scored higher than bvFTD

MANOVAs showed significant differences among the groups on the FACT [Pillai’s trace 0.9, *F*(27,165) = 2.62], FCAI-6 [Pillai’s trace 0.84, *F*(18,174) = 3.78] and FCAI-4 legal scales [Pillai’s trace = 0.84, *F*(12,177) = 5.76], *p*s < 0.001. This result was replicated using a multivariate analysis of covariance in which age was the covariate.

**Fig. 1:**
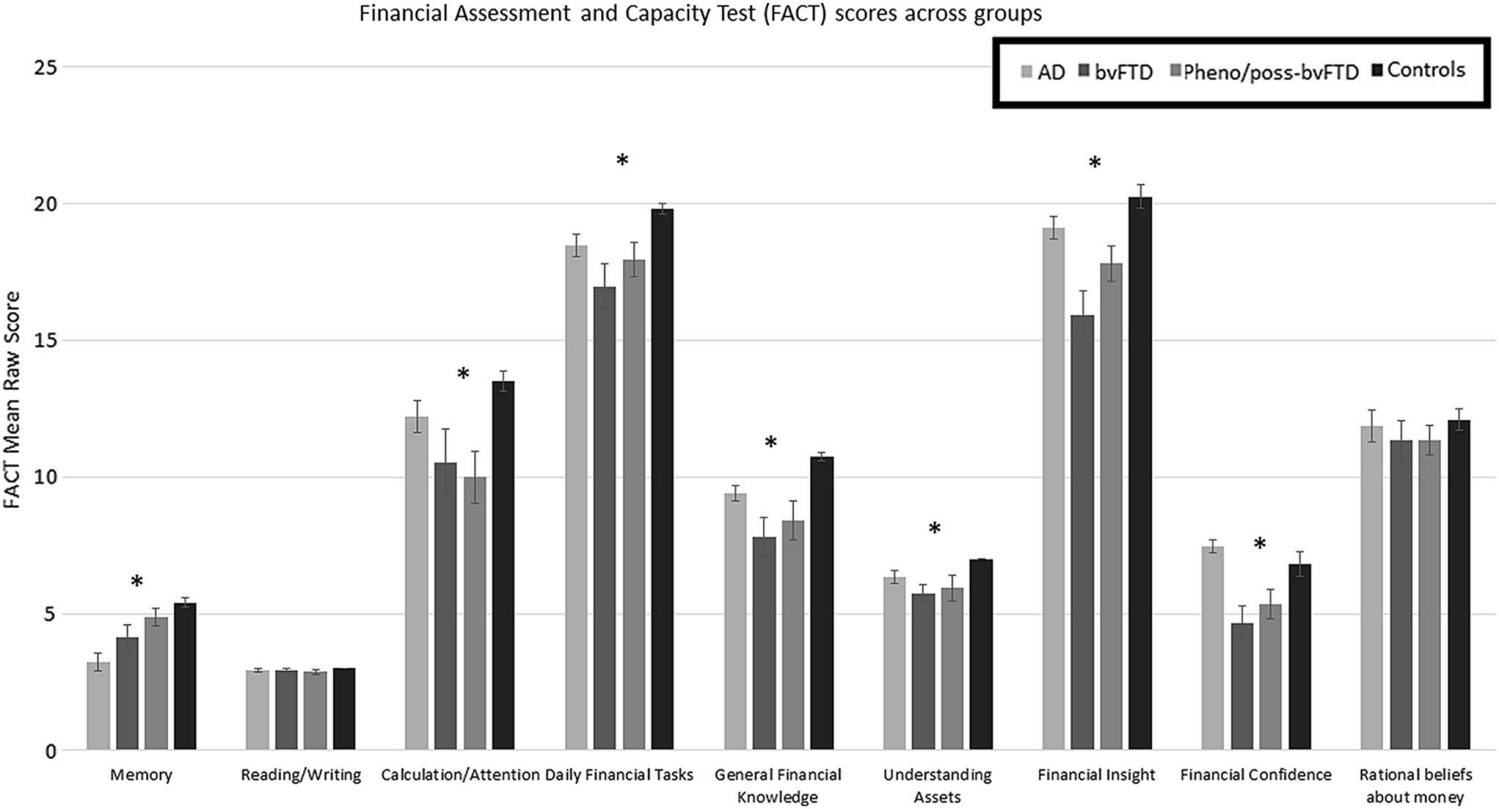
Comparison of mean FACT raw scores across groups. Asterisk: significant findings using one-way ANOVAs with Bonferroni-corrected post hoc contrasts. Memory: controls and pheno/poss-bvFTD scored higher than AD; controls scored higher than bvFTD. Calculation/Attention: controls scored higher than pheno/poss-bvFTD. Daily Financial Tasks: controls scored higher than bvFTD. General Financial Knowledge: controls scored higher than pheno/poss-bvFTD and bvFTD. Understanding Assets: controls scored higher than bvFTD. Financial Insight: controls and AD scored higher than bvFTD; controls scored higher than pheno/poss-bvFTD. Financial Confidence: controls scored higher than bvFTD; AD scored higher than pheno/poss-bvFTD and bvFTD. FACT total score (not shown): controls scored higher than pheno/poss-bvFTD and bvFTD; AD scored higher than bvFTD

Univariate ANOVAs demonstrated significant group differences on most FACT scales (with the exception of Reading/Writing and Rational Beliefs about Money) and the total FACT score, *p*s < 0.05 (see Fig. 1; supplementary Table 2). On post hoc analyses, bvFTD patients scored lower than healthy volunteers on Memory, Daily Financial Tasks, General Financial Knowledge, Understanding Assets, Financial Insight, and Financial Confidence scales of the FACT, *p*s< 0.05. In addition, patients with bvFTD scored lower than patients with AD on FACT scales of Financial Insight and Financial Confidence scales, the latter of which examines current financial difficulties (e.g., money problems, strain in relationship due to money), *p*s< 0.05 (see Fig. 1; supplementary Table 2). A significant difference between bvFTD and AD groups on the FACT Total score, *p* = 0.03, was no longer significant when age was controlled for using ANCOVA, *p* = 0.2. The pheno/poss-bvFTD group scored poorly compared to healthy controls on FACT scales of Calculation/Attention, General Financial Knowledge, and Financial Insight, and FACT total, *p*s < 0.05, and lower than AD patients on Financial Confidence.

**Fig. 2:**
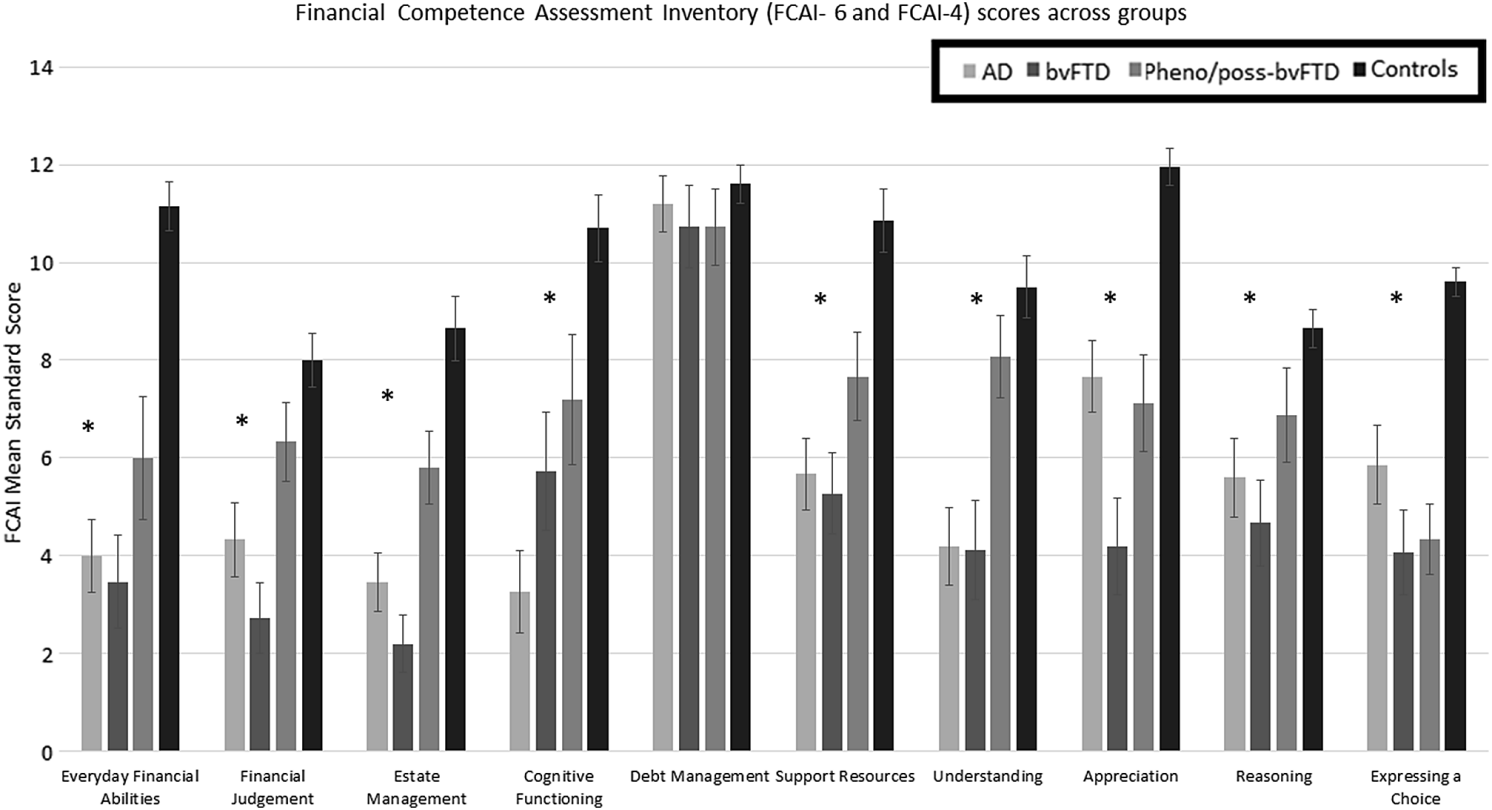
Comparison of mean FCAI standard score across groups. Asterisk: significant findings using one-way ANOVAs with Bonferroni-corrected post hoc contrasts: Everyday Financial Abilities: controls scored higher than pheno/poss-bvFTD, bvFTD and AD. Financial Judgement: controls scored higher than AD and bvFTD; pheno/poss-bvFTD scored higher than bvFTD. Estate Management: controls scored higher than pheno/poss-bvFTD, AD and bvFTD; pheno/poss-bvFTD scored higher than bvFTD. Cognitive Functioning: controls scored higher than bvFTD and AD. Support Resources: controls scored higher than pheno/poss-bvFTD, AD and bvFTD. Understanding: controls and pheno/poss-bvFTD scored higher than AD and bvFTD. Appreciation: controls scored higher than AD, pheno/poss-bvFTD and FTD; AD scored higher than bvFTD. Reasoning: controls scored higher than AD and bvFTD. Expressing a Choice: controls scored higher than AD, pheno/poss-bvFTD and bvFTD. FCAI total score (not shown): controls scored higher than pheno/poss-bvFTD, AD and bvFTD

FCAI total scores fell in the impaired range (more than one standard deviation below the mean) for 86% of the patients with FTD, 93% of the patients with AD, and 66% of the patients with pheno/poss-bvFTD in comparison to 10% of the age-matched controls. In regard to the FCAI-6 and FCAI-4, one-way ANOVAs showed significant group differences on most scales, *p*s < 0.05 (see Fig. 2; supplementary Tables 3 and 4), except for debt management, *p *=* 0*.72 (see supplementary Table 3). Post hoc analyses revealed poor scores across most scales by patients with bvFTD (and the other clinical groups, AD and pheno/poss-bvFTD) compared to the healthy controls, *p*s < 0.05 (see Fig. 2; supplementary Tables 3 and 4). The bvFTD group had lower scores compared to the AD group on the FCAI Appreciation Scale, *p* = 0.02, indicating that bvFTD patients had more difficulty appreciating the consequence of their decisions; however, this difference was no longer significant when age was taken into account using ANCOVA, *p* = 0.1. The pheno/poss-bvFTD group scored poorly compared to healthy controls on FCAI scales of Everyday Financial Abilities, Estate Management, Support Resources, Appreciation, and Expressing a Choice, and also the FCAI Total score, *p*s < 0.05 (see Fig. 2; supplementary Tables 3 and 4).

We should note that homogeneity and normality assumptions were not met for all ANOVAs performed. The homogeneity assumption (examined with Levene’s test) was met for two of the nine subscales of the FACT, four of the six subscales of the FCAI-6, and one of the four subscales of the FCAI-4. The normality assumption (tested with Shapiro–Wilk for each group) was met for as many as three of the nine subscales of the FACT, four subscales of the FCAI-6, and three subscales of the FCAI-4. Thus, to complement the aforementioned analyses, we also conducted non-parametric tests, namely Kruskal–Wallis *H* test and Mann–Whitney *U* tests, with Bonferroni post hoc contrasts to correct for multiple comparisons. The pattern of results replicated the aforementioned results on the FACT, FCAI-6 and FCAI-4 tests, with few exceptions (see supplementary Table 7), most notably, significantly worse performance by AD patients relative to healthy controls on FACT scales of Daily Financial Tasks, General Financial Knowledge, Understanding Assets, and FACT Total score.

**Fig. 3:**
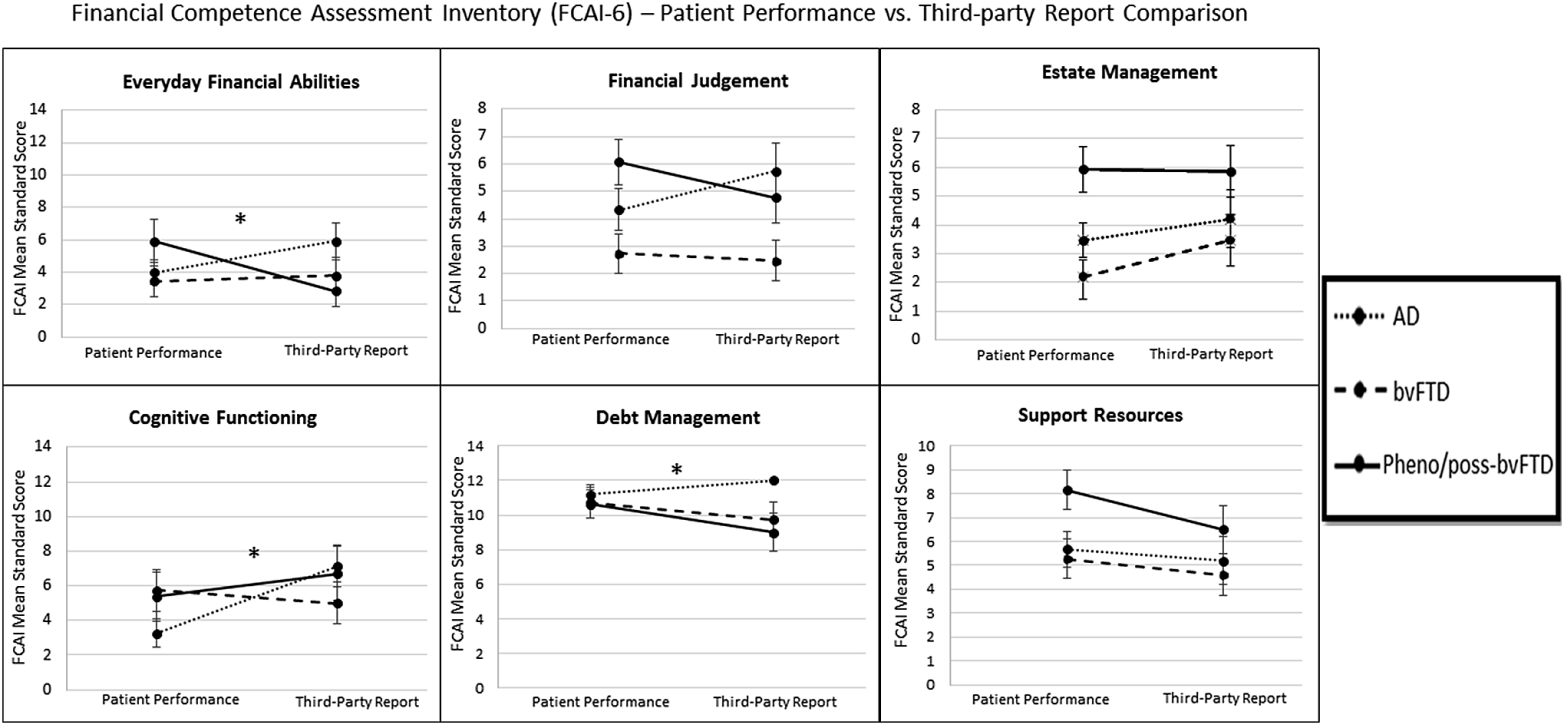
Financial Competence Assessment Inventory (FCAI-6) patient performance and caregiver report for the six domains. All results indicated are based on mixed-factorial ANOVAs, *p*s < 0.05. Asterisk: significant interactions

**Fig. 4:**
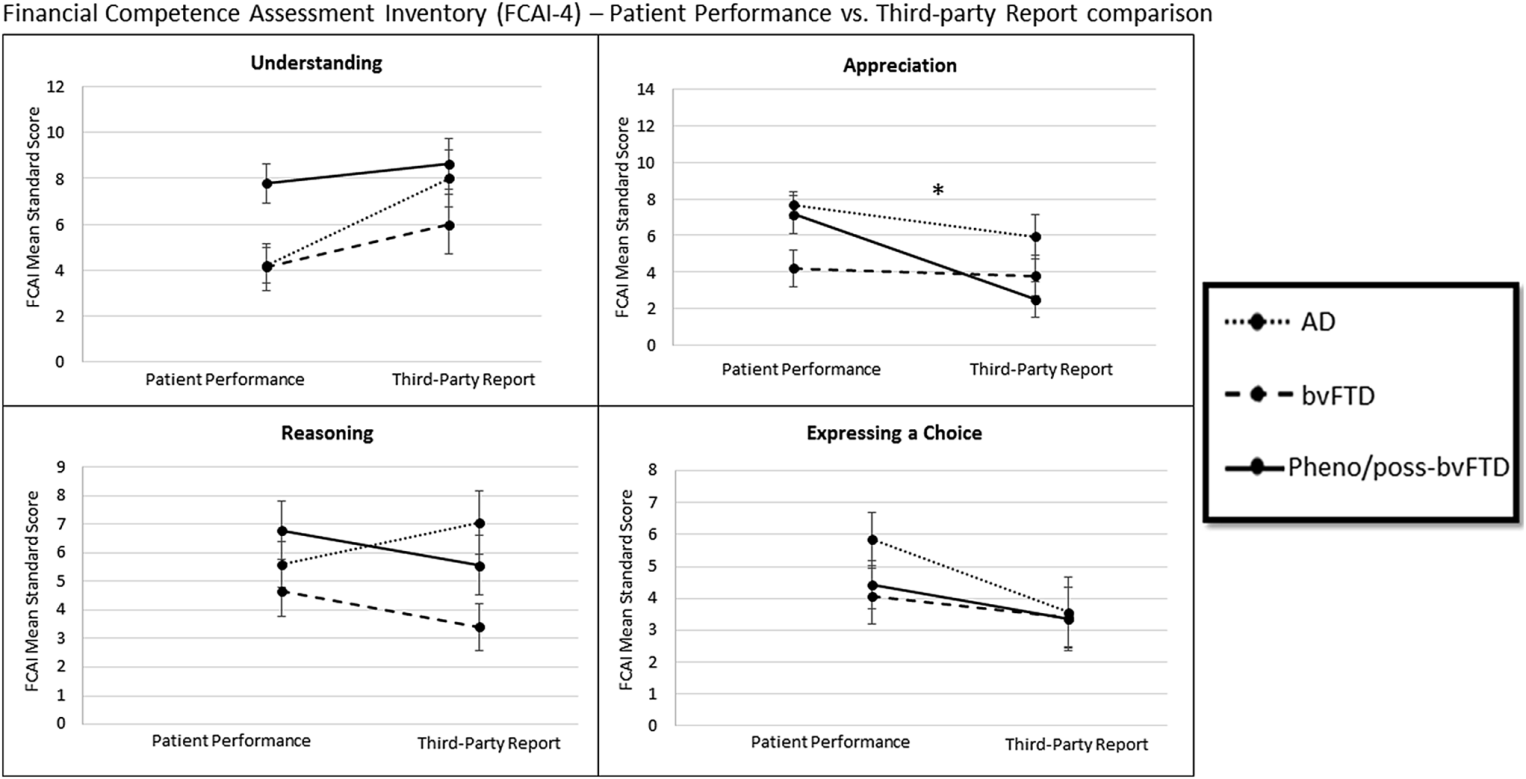
Financial Competence Assessment Inventory (FCAI-4) patient performance and caregiver report for the four domains. All results indicated are based on mixed-factorial ANOVAs, *p*s < 0.05. Asterisk: significant interactions

Figures 3 and 4 show the results of Group (bvFTD, pheno/poss-bvFTD, AD) × FCAI-format (patient performance, third-party report) mixed-factorial ANOVAs (see supplementary Tables 5 and 6). As demonstrated, results on scales of Everyday Financial Abilities, Cognitive Functioning, Debt Management, and Appreciation were qualified by significant interactions, *p*s < 0.05. Post-hoc analyses using paired *t* tests showed only a significant result for AD patients on cognitive functioning, *t*(14) = 3.4, *p* = 0.004 (using *α* level of 0.017 based on Bonferroni adjustment for multiple comparisons across three groups); as shown in Fig. 3, third-party reports significantly overestimated AD patients cognitive skills on the FCAI Cognitive Functioning scale. The results of these discrepancy analyses between clinical group scores and third-party reports were generally in line with the results of a mixed-factorial ANCOVA (age as covariate) and a non-parametric approach (Wilcoxon signed-rank test), in which significance criteria was either achieved or were at marginal levels (with Bonferroni applied). Thus, the aforementioned findings based on these discrepancy analyses should be interpreted cautiously.

## Discussion

Patients presenting to their physician with changes in financial judgement represent a diagnostic and management challenge. While impairment in financial abilities has been characterized in MCI and AD, how patients with possible or probable FTD perform on validated financial assessments is unclear. As the diagnosis of bvFTD is based on an early onset of behavioural and executive functional deficits, financial capacity is an imperative functional skill that should be clinically assessed and monitored. The younger age of patients with FTD makes them more likely to be active in handling money and making financial decisions both for themselves and their families. While clinicians often identify cases of financial incompetence based on history [7], objective measure of financial abilities in FTD has been largely unexplored. In line with predictions, bvFTD patients demonstrated poor financial abilities relative to healthy controls; and relative to patients with AD with similar overall cognitive functioning, patients with bvFTD scored poorly in financial domains of insight and confidence. Thus, our results emphasize the importance of evaluating financial capacity in individuals with suspected bvFTD as they tend to show impairment on several financial domains. Additionally, discrepancy between patient and caregiver results, particularly in AD patients, highlights the inherent subjective nature of third-party reports and suggests caution in interpreting such reports.

While there is evidence of impaired financial functioning in bvFTD through subjective reporting or indirect assessments of daily living [14], to date only one recently published study employed a performance-based measure to examine financial abilities in FTD [29]. Using a financial assessment tool developed in Greece, Giannouli et al. [29], observed that bvFTD patients performed significantly worse than the healthy participants. Their results are generally consistent with our findings, which involved an English-speaking cohort of bvFTD patients who demonstrated difficulties in performing financial tasks. Going beyond Giannouli et al.’s work [29], an added value of the present study is the use of two validated measures and the comparison of patient performance with third-party reports to elaborate on the financial capacity in this population.

In our study, the overall performance of bvFTD patients was worse than that of AD patients on one of the two financial measures. The bvFTD group performed particularly worse than AD patients on the FACT scales of Financial Insight and Financial Confidence. With respect to financial confidence, it should be noted that this scale is based on identifying self-reported current financial difficulty; thus, the higher scores by AD patients are not surprising, as it may not actually reflect good financial standing and instead highlight poor recall/inability to identify current financial issues. With regard to poor financial insight in bvFTD, this finding may reflect a general pattern of poor insight into cognitive and behavioural changes in this population [6]. Moreover, bvFTD patients’ low financial insight is in line with their poor performance on the FCAI Appreciation scale relative to AD patients, a scale that examines the ability to think abstractly and appreciate the consequences and implications of financial decisions [17]. Considered together, the financial capacity deficits identified in bvFTD patients relative to AD patients likely relate to lack of insight, inability to think in an abstract manner about the consequences of one’s decision, and overall executive function deficits well known in bvFTD patients.

It is typically family members who raise concerns about a change in patients’ financial judgement or abilities. The clinical management of such patients who do not yet meet criteria for a diagnosis of dementia represents a significant challenge, particularly as patients and family members’ reports of such incidents are frequently discrepant. We found that in comparison to their performance on the FACT and FCAI, study partners showed a general tendency to underestimate the financial abilities of patients with possible FTD. However, as a group, patients with pheno/poss-bvFTD performed poorly on both the FACT and the FCAI compared to controls, supporting the clinical utility of either of these tools for objective assessments of financial ability in patients with possible FTD. Caregivers of patients with bvFTD tended to under- and over-estimate patients’ financial abilities as assessed by the FCAI, but these differences did not reach significance on post hoc analyses. In contrast, caregivers of patients with AD tended to over-estimate patients’ financial abilities on the FCAI. A significant difference was found between caregivers and AD patients on the FCAI Cognitive Functioning scale, but this finding should be interpreted cautiously because although borne out with non-parametric analysis, only a marginal level finding was noted when age differences across the groups were taken into account. In contrast to patients with bvFTD who have reduced insight and difficulties with poor judgement and impulse control, it is known that people with early AD can often hide cognitive symptoms (e.g., refusing to participate in an activity or using strategies to deal with poor memory) so as to maintain independence. Caregivers of patients with early AD may not be fully aware of the person’s impairment/limitations in managing finances. The lack of difference in performance and estimates in the bvFTD group is of interest, as in early stages of bvFTD, caregivers may note many deficits in everyday activities, yet patient performance on standard cognitive testing may be preserved. Indeed this was a motivation for the present study, in which we hypothesized that financial cognitive tests, which are more closely aligned to real-world behaviours, require judgement as well as valuations, might reveal deficits. The lack of a significant difference between bvFTD performance and caregiver estimates may reflect sensitivity of these tests to cognitive deficits in bvFTD, as this group performed poorly. Whether patient performance on the financial assessments would be intact relative to caregiver estimates at the earliest stages of bvFTD, such as we observed in the pheno/poss-bvFTD group, remains to be confirmed in a cohort with mild probable bvFTD.

There are a limited number of performance-based measures to assess financial capacity in clinical populations. The FACT and FCAI were selected because of their validation in multiple psychogeriatric populations [16, 17]. While financial deficits were identified in all clinical groups using both scales in this study, in the case of time constraints, the FACT scale as a whole and the FCAI legal scales appear sufficient to detect impairments. Moreover, to discriminate between the bvFTD and AD populations, we recommend close examination of the FACT Insight scale and the FCAI Appreciation scale, which reduces the evaluation time by half and may be a beneficial battery in clinical assessments where time is constrained. While the AD and bvFTD groups were diagnosed based on clinical criteria, further validation with larger samples, and comparison of autopsy confirmed AD and bvFTD will be helpful to confirm the patterns observed in this clinical cohort. Moreover, sex differences limit the results of the study, which largely stemmed from a higher proportion of males in the pheno/possible bvFTD group.

In conclusion, this research highlights financial difficulties in individuals with bvFTD and provides new evidence from established performance-based measures that are validated to detect such deficits in clinical populations. As clinicians are often required to address questions of financial abilities and decision-making capacity, the outcomes of formal measures (such as the FACT and FCAI) can complement subjective reports to help clinicians make well informed decisions, and should be interpreted within the context of the patient’s functional history (e.g., whether the individual has always done his/her own finances).

## Electronic supplementary material

Below is the link to the electronic supplementary material.

Supplementary material 1 (DOCX 49 kb)
