# Supplementary material for: Financial capacity in frontotemporal dementia and related presentations
Source: J Neurol. 2019 Apr 22;266(7):1698–707. doi: 10.1007/s00415-019-09317-w (PMC6586696; doi:10.1007/s00415-019-09317-w)
Supplement: Supplementary file 1 — Supplementary material 1 (DOCX 49 kb) [file 415_2019_9317_MOESM1_ESM.docx]

**Supplemental Table 1:** Demographics, clinical characterization and follow-up of the possible FTD/ FTD phenocopy group

| **Age** | **Sex** | **Initial diagnosis** | **Financial symptoms at presentation** | **Secondary symptoms** | **Neuroimaging** | **Diagnosis at follow up (duration of follow up)** |
| --- | --- | --- | --- | --- | --- | --- |
| 73 | M | Pheno/poss-bvFTD | Previously successful businessman. In debt tens of thousands of dollars from multiple poor investment decisions. Buying redundant appliances, scooters, furniture despite filing for bankruptcy. | Decreased empathy, hypersexuality, reduced personal hygiene, irritability | MRI mild generalized atrophy | Probable FTD (3y): MRI with progressive frontal and temporal atrophy |
| 64 | M | Pheno/poss- bvFTD | Excessive buying of shirts. | Disinhibition, increased anger, apathy | MRI wnl.  SPECT: rt temporal, ACC and PCC hypoperfusion | Depression, intermittent explosive disorder, cluster B personality traits, stable MRI (4 y) |
| 64 | M | Pheno/poss-bvFTD | Impulsive purchases, spending more money on food. | Apathy, hypersexual, disinhibition | Mild peri-sylvian atrophy; SPECT: mild reduction to bifrontal and anterior temporal | bvFTD based on symptom progression and progressive FT atrophy (4y) |
| 71 | M | Pheno/poss-bvFTD | Impulsive spending (purchased 3 cruises in 1 day). | Empathy deficits, irritability, apathy, hoarding, personal neglect | No focal atrophy | Depression, improved (8 y) |
| 78 | M | Pheno/poss-bvFTD vs. frontal AD | 2 years of impulsive financial decisions. | Hyperorality, inattention, irritability, possible confabulation, mild disinhibition | Old cerebellar infarcts | VCI; CT 2016 new right thalamic, internal capsule infarctions |
| 76 | F | Pheno/poss-bvFTD | Impulsive real estate decisions. | Social apathy, compulsive behaviours | Bifrontal atrophy | Possible bvFTD vs. phenocopy (1y) |
| 68 | M | Pheno/poss-bvFTD | Spent $20,000 of $150,000 retirement savings on drinking and smoking over 2 years. | Disinhibition, inappropriate social comments, irritability, apathy | Normal MRI | Suspected longstanding autistic spectrum traits in setting of recent second marriage (1y) |
| 64 | M | Pheno/poss-bvFTD | Impulsive purchases, new lack of awareness of finances. | Inappropriate jocularity, word finding difficulty, impulsivity, executive function deficits | Normal MRI; normal SPECT | Possible bvFTD vs. phenocopy (2y) |
| 62 | M | Pheno/poss-bvFTD | Spending $100,000s including retirement savings towards irrational business plan. | Hypersexuality, impulsivity, poor judgement, | Normal MRI, normal SPECT | Possible bvFTD vs. phenocopy (1 y) |
| 72 | M | Pheno/poss-bvFTD | None specific- spouse always handled finances. | Memory deficits, later chorea, followed by reduced empathy | Rt. temporal pole atrophy, mild diffuse frontal atrophy b/l, small lentiform hyperintensity | Auto-immune limbic encephalitis (3y) |
| 69 | M | Pheno/poss-bvFTD | Questionable impulsive purchases. | Executive function changes, irritability, apathy | MRI normal | depression, relationship conflict (2y) |
| 79 | M | MCI: possible frontal AD vs. Pheno/poss-bvFTD | Impulsive purchasing- computer, I-phone, new car | Mild memory and executive function deficits, irritability, disinhibition, apathy | Punctate right parietal infarct, otherwise normal | Probable Frontal AD; MRI with diffuse atrophy (6y) |
| 72 | M | Pheno/poss-bvFTD | Impulsive spending | Irritability, semantic paraphasias, inappropriate jocularity, disinhibition | No focal atrophy on MRI, normal SPECT | EtOH abuse, cluster B personality traits (2 year) |
| 58 | M | Pheno/poss-bvFTD | Stopped paying bills, $5000 overdue | Absentminded, forgetting to pick up family at airport, not showing up for work meetings | Mild bifrontal and temporal atrophy on MRI, SPECT:  frontal and temporal hypoperfusion | Did not return for follow up (poss-bvFTD based on lack of follow up to confirm progression) |
| 79 | M | Frontal AD vs. Pheno/poss-bvFTD | Frequent on-line stock trading but no major issues at presentation | disinhibition, socially inappropriate comments, distractible, hoarding, impulsive, restlessness, irritability, short term memory deficits | Normal CT | Frontal variant AD: MRI bitemporal atrophy with hippocampal atrophy (1y) |

**Supplemental Table 2:** Financial Assessment and Capacity Test (FACT) raw score comparisons across the groups

| FACT Scales  (Maximum score) | AD  (n = 15)  *M* (SD) | bvFTD  (n = 15)  *M* (SD) | Pheno/poss-bvFTD  (n = 15)  *M* (SD) | Controls  (n = 20)  *M* (SD) | *F*-value | df | *p*-value | *η^2^_p_* |
| --- | --- | --- | --- | --- | --- | --- | --- | --- |
| Memory (6) | 3.23 (1.29) | 4.13(1.73) | 4.87(1.30) | 5.40(.82) | 8.85 | (3, 61) | ^a^.000 | .30 |
| Reading/Writing (3) | 2.93(.26) | 2.93(.26) | 2.87(.35) | 3.00(0.00) | .87 | (3, 61) | .462 | .04 |
| Calculation/Attention (14) | 12.20(2.21) | 10.53(4.69) | 10.0(3.70) | 13.5(1.67) | 4.33 | (3, 61) | ^b^.008 | .18 |
| Daily Financial Tasks (21) | 18.47(1.64) | 16.97(3.14) | 17.93(2.43) | 19.80(0.83) | 5.50 | (3,61) | ^c^.002 | .21 |
| General Financial Knowledge (11) | 9.40(1.12) | 7.80(2.81) | 8.40(2.72) | 10.75(.64) | 7.45 | (3, 61) | ^d^.000 | .27 |
| Understanding Assets (7) | 6.33(.90) | 5.73(1.28) | 5.93(1.87) | 7.00(0.00) | 4.068 | (3,61) | ^e^.011 | .17 |
| Financial Insight (24) | 19.13(1.60) | 15.93(3.47) | 17.80(2.54) | 20.25(1.97) | 9.54 | (3,61) | ^f^.000 | .32 |
| Financial Confidence (8) | 7.47(.92) | 4.67(2.35) | 5.33(2.09) | 6.80(1.99) | 6.98 | (3, 61) | ^g^.000 | .26 |
| Rational beliefs about money (14) | 11.87 (2.20) | 11.33(2.79) | 11.33(2.09) | 12.10(1.77) | .52 | (3,61) | .67 | .03 |
| FACT total (108) | 90.37(7.41) | 79.70(15.36) | 84.40(11.13) | 98.60(4.95) | 11.30 | (3, 61) | ^h^ 0.00 | 0.36 |

*Note.* AD = Alzheimer’s disease; bvFTD = Behavioural variant - Frontotemporal dementia; *M* = mean; Pheno/poss-bvFTD = phenocopy or possible behavioural variant Frontotemporal dementia; SD = standard deviation

All results indicated are based on univariate ANOVAs, *ps <*0.05.

^a^ Controls & pheno/poss-bvFTD scored higher than AD; Controls scored higher than bvFTD

^b^ Controls scored higher than pheno/poss-bvFTD

^c^ Controls scored higher than bvFTD

^d^ Controls scored higher than pheno/poss-bvFTD & bvFTD

^e^ Controls scored higher than bvFTD

^f^ Controls & AD scored higher than bvFTD; Controls scored higher than pheno/poss-bvFTD

^g^ Controls scored higher than bvFTD; AD scored higher than pheno/poss-bvFTD & bvFTD

^h^ Controls scored higher than pheno/poss-bvFTD & bvFTD; AD scored higher than bvFTD

**Supplemental Table 3:** Financial Competence Assessment Inventory (FCAI-6) standard score comparisons across the groups for the 6 domains

| FCAI Scales  (Maximum Standard Score) | AD  (n = 15)  *M* (SD) | bvFTD  (n = 15)  *M* (SD) | Pheno/poss-bvFTD  (n = 15)  *M* (SD) | Controls  (n = 20)  *M (*SD) | *F*-value | df | *p*-value | *η^2^_p_* |
| --- | --- | --- | --- | --- | --- | --- | --- | --- |
| Everyday Financial Abilities (14) | 4.00(2.88) | 3.47(3.66) | 6.00(4.88) | 11.15(2.23) | 18.55 | (3, 61) | ^a^.000 | .48 |
| Financial Judgement (14) | 4.33(2.92) | 2.73(2.79) | 6.33(3.13) | 8.0(2.45) | 11.47 | (3, 61) | ^b^.000 | .36 |
| Estate Management (14) | 3.47(2.33) | 2.20(2.27) | 5.80(2.88) | 8.65(2.91) | 20.22 | (3, 61) | ^c^ .000 | .50 |
| Cognitive Functioning (13) | 3.27(3.26) | 5.733(4.68) | 7.20(5.17) | 10.70(3.03) | 10.27 | (3, 61) | ^d^.000 | .34 |
| Debt Management (12) | 11.20(2.24) | 10.73(3.39) | 10.73(3.03) | 11.60(1.79) | .44 | (3, 61) | .723 | .02 |
| Support Resources (14) | 5.67(2.87) | 5.27(3.22) | 7.67(3.50) | 10.85(2.87) | 12.07 | (3, 61) | ^e^.000 | .37 |
| FCAI total (125) | 72.47(5.21) | 72.73(7.90) | 78.70(13.00) | 98.45(14.42) | 22.09 | (3. 61) | ^f^.00 | .52 |

*Note.* AD = Alzheimer’s disease; bvFTD = Behavioural variant - Frontotemporal dementia; *M* = mean; pheno/poss-bvFTD = phenocopy or possible behavioural variant Frontotemporal dementia; SD = standard deviation

All results indicated are based on univariate ANOVAs, *ps <*0.05.

^a^ Controls scored higher than pheno/poss-bvFTD, bvFTD & AD

^b^ Controls scored higher than AD & bvFTD; pheno/poss-bvFTD scored higher than bvFTD

^c^ Controls scored higher than pheno/poss-bvFTD, AD & bvFTD; pheno/poss-bvFTD scored higher than bvFTD

^d^ Controls scored higher than bvFTD & AD

^e^ Controls scored higher than pheno/poss-bvFTD, AD & bvFTD

^f^ Controls scored higher than pheno/poss-bvFTD, AD & bvFTD

**Supplemental Table 4:** Financial Competence Assessment Inventory (FCAI-4) standard score comparisons across the groups for the 4 legal domains.

| FCAI Scales  (Maximum Standard Score) | AD  (n = 15)  *M* (SD) | bvFTD  (n = 15)  *M* (SD) | Pheno/poss-bvFTD  (n = 15)  *M* (SD) | Controls  (n = 20)  *M* (SD) | *F*-value | df | *p*-value | *η^2^_p_* |
| --- | --- | --- | --- | --- | --- | --- | --- | --- |
| Understanding (13) | 4.20 (3.05) | 4.13 (3.93) | 8.07 (3.28) | 9.50 (2.87) | 11.73 | (3,61) | ^a^ .000 | .37 |
| Appreciation (13) | 7.67 (2.87) | 4.20 (3.82) | 7.13 (3.83) | 11.95 (1.70) | 18.96 | (3,61) | ^b^.000 | .48 |
| Reasoning (13) | 5.60 (3.11) | 4.67 (3.37) | 6.87 (3.74) | 8.65 (1.76) | 5.78 | (3,61) | ^c^.002 | .22 |
| Expressing a choice (11) | 5.86 (3.11) | 4.07 (3.37) | 4.33 (2.79) | 9.60 (1.31) | 16.55 | (3,60) | ^d^.000 | .45 |

*Note.* AD = Alzheimer’s disease; bvFTD = Behavioural variant - Frontotemporal dementia; *M* = mean; pheno/poss-bvFTD = phenocopy or possible behavioural variant Frontotemporal dementia; SD = standard deviation

All results indicated are based on univariate ANOVAs, *ps <*0.05.

^a^ Controls & pheno/poss-bvFTD scored higher than AD & bvFTD

^b^ Controls scored higher than AD, pheno/poss-bvFTD & FTD; AD scored higher than bvFTD

^c^ Controls scored higher than AD & bvFTD

^d^ Controls scored higher than AD, pheno/poss-bvFTD & bvFTD

**Supplemental Table 5:** Financial Competence Assessment Inventory (FCAI-6) patient performance and caregiver report comparisons results for the 6 domains

| Patient – Third-party Score comparison | Between Subjects Main Effect  *F*(df), *p,* *η^2^_p_* | Within Subjects Main Effect *F*(df), *p,* *η^2^_p_* | Interaction  *F*(df), *p,* *η^2^_p_* |
| --- | --- | --- | --- |
| Everyday Financial Abilities | 0.59(2, 41), 0.56, 0.03 | 0.16(1, 41), 0.70, 0.00 | 4.80(2, 41), 0.01, 0.19^c^ |
| Financial Judgement | 5.02(2, 41), 0.01, 0.20 ^a^ | 0.01(1, 41), 0.93, 0.00 | 1.85(2, 41), 0.17, 0.08 |
| Estate Management | 4.76(2, 41), 0.01, 0.19 ^b^ | 2.08(1, 41), 0.16, 0.05 | 0.75(2,41), 0.48, 0.04 |
| Cognitive Functioning | 0.85(2, 41), 0.44, 0.04 | 1.25(1, 41), 0.27, 0.03 | 3.68(2, 41), 0.03, 0.15^d^ |
| Debt Management | 1.54(2, 41), 0.23, 0.07 | 2.79(1, 41), 0.10, 0.06 | 3.96(2, 41), 0.03, 0.16^e^ |
| Support Resources | 3.42(2, 41), 0.04, 0.14 | 2.01(1, 41), 0.16, 0.05 | 0.30(2, 41), 0.74, 0.02 |
| FCAI total | 0.41(2, 41), 0.67, 0.02 | 0.11(1, 41), 0.74, 0.003 | 2.93(2, 41), 0.07, 0.13 |

*Note*. All results indicated are based on mixed-factorial ANOVA, *ps* <0.05.

^a^ AD and pheno/poss-bvFTD scores are higher than bvFTD scores (See Figure 3)

^b^ pheno/poss-bvFTD scores are higher than AD scores (See Figure 3)

^c,d,e^ Significant interactions illustrated in Figure 3

**Supplemental Table 6:** Financial Competence Assessment Inventory (FCAI-4) patient performance and caregiver report comparison results for the 4 legal domains.

| Patient – Third-party Score comparison | Between Subjects Main Effect  *F*(df), *p,* *η^2^_p_* | Within Subjects Main Effect F, *F*(df), *p,* *η^2^_p_* | Interaction  *F*(df), *p,* *η^2^_p_* |
| --- | --- | --- | --- |
| Understanding | 3.47(2, 41), 0.04, 0.15^a^ | 8.41(1, 41), 0.01, 0.17^b^ | 1.32(2, 41), 0.28, 0.06 |
| Appreciation | 2.97(2, 41), 0.06, 0.13 | 11.73(1, 41), 0.001, 0.22^c^ | 3.54(2, 41), 0.04, 0.15^e^ |
| Reasoning | 2.80(2, 41), 0.07, 0.12 | 0.28(1, 41), 0.60, 0.01 | 1.99(2, 41), 0.15, 0.09 |
| Expressing a Choice | 0.50(2, 40), 0.61, 0.02 | 4.46(1, 40), 0.04, 0.1 ^d^ | 0.59(2, 40), 0.56, 0.03 |

*Note*. All results indicated are based on mixed-factorial ANOVAs, *ps* <0.05.

^a^ pheno/poss-bvFTD scores are higher than bvFTD scores (See Figure 3)

^b^ Patient scores are lower than Third party scores (See Figure 3)

^c,d^ Patient scores are higher than Third party scores (See Figure 3)

^e^ Significant interactions shown in Figure 3

**Supplemental Table 7:** Financial Assessment and Capacity Test (FACT) raw score comparisons and Financial Competence Assessment Inventory (FCAI-6 & FCAI-4) standard score comparisons across the groups based on non-parametric analyses

|  | Kruskal Wallis  H | *df* | *p*-value | Post hoc results using Mann-Whitney U Test (Bonferroni corrected, *ps* < .008) |
| --- | --- | --- | --- | --- |
| **FACT Scales** |  |  |  |  |
| Memory | 20.51 | 3 | <.001 | Controls & pheno/poss-bvFTD scored higher than AD |
| Reading/Writing | 2.623 | 3 | 0.45 |  |
| Calculation/Attention | 13.17 | 3 | 0.004 | Controls scored higher than pheno/poss-bvFTD & bvFTD |
| Daily Financial Tasks | 13.55 | 3 | 0.004 | Controls scored higher than AD & pheno/poss-bvFTD |
| General Financial Knowledge | 24.45 | 3 | <.001 | Controls scored higher than AD, pheno/poss-bvFTD & bvFTD |
| Understanding Assets | 16.49 | 3 | 0.001 | Controls scored higher than AD & bvFTD |
| Financial Insight | 16.91 | 3 | 0.001 | Controls scored higher than pheno/poss-bvFTD & bvFTD |
| Financial Confidence | 16.55 | 3 | 0.001 | Controls scored higher than bvFTD; AD scored higher than pheno/poss-bvFTD & bvFTD |
| Rational beliefs about money | 1.24 | 3 | 0.74 |  |
| FACT total | 24.58 | 3 | <.001 | Controls scored higher than AD, pheno/poss-bvFTD & bvFTD |
|  |  |  |  |  |
| **FCAI Scales** |  |  |  |  |
| Everyday Financial Abilities | 29.74 | 3 | <.001 | Controls scored higher than AD, pheno/poss-bvFTD & bvFTD |
| Financial Judgement | 22.51 | 3 | <.001 | Controls scored higher than AD & bvFTD; pheno/poss-bvFTD scored higher than bvFTD |
| Estate Management | 32.64 | 3 | <.001 | Controls scored higher than AD & bvFTD; pheno/poss-bvFTD scored higher than bvFTD |
| Cognitive Functioning | 21.06 | 3 | <.001 | Controls scored higher than AD & bvFTD |
| Debt Management | 1.72 | 3 | 0.63 |  |
| Support Resources | 22.90 | 3 | <.001 | Controls scored higher than AD & bvFTD |
| FCAI total | 37.12 | 3 | <.001 | Controls scored higher than AD, pheno/poss-bvFTD & bvFTD |
| Understanding | 23.22 | 3 | <.001 | Controls & poss-bvFTD scored higher than AD & bvFTD |
| Appreciation | 31.68 | 3 | <.001 | Controls scored higher than AD, pheno/poss-bvFTD & bvFTD |
| Reasoning | 14.40 | 3 | 0.002 | Controls scored higher than AD & bvFTD |
| Expressing a choice | 30.29 | 3 | <.001 | Controls scored higher than AD, pheno/poss-bvFTD & bvFTD |

*Note.* AD = Alzheimer’s disease; bvFTD = Behavioural variant - Frontotemporal dementia; pheno/poss-bvFTD = phenocopy or possible behavioural variant Frontotemporal dementia
